# Exploring and Validating Prognostic Biomarkers Related to Sphingolipid Metabolism in Gastric Cancer through Machine Learning

**DOI:** 10.2174/0118715303362774250219065927

**Published:** 2025-03-26

**Authors:** Jian Chai, Ce Guo, Houze Wang, Jiajie Wei, Yang Yu, Xiaolong LI, Huiqing Zhang, Xing Guo

**Affiliations:** 1 Chengde Medical College, Chengde City, Hebei Province, China;; 2 Department of General Surgery, Baoding First Central Hospital, Baoding City, Hebei Province, China;; 3 Baoding Key Laboratory of Gastrointestinal Tumor Diagnosis and Treatment, Baoding City, Hebei Province, China;; 4 Department of Oncology, Baoding First Central Hospital, Baoding City, Hebei Province, China

**Keywords:** Bioinformatics, machine learning, gastric cancer, sphingolipid metabolism, prognosis, immunohistochemistry

## Abstract

**Background:**

Sphingolipid metabolism (SM) has been implicated in the progression of gastric cancer (GC). However, its potential as a prognostic biomarker in GC remains underexplored. This study investigates the feasibility of using SM to predict GC prognosis.

**Methods:**

Sphingolipid metabolism-related genes (SMRGs) were extracted from the GeneCards database, and differentially expressed genes (DEGs) were identified using the TCGA-STAD and GSE84437 gastric cancer datasets. Univariate Cox regression analysis was performed to identify genes associated with survival. Lasso-Cox and random survival forest analyses were employed to identify key survival-related genes, followed by multivariate Cox regression to establish a prognostic model and calculate the sphingolipid metabolism score (SMscore). The lasso-Cox analysis further assessed the prognostic significance of clinical traits and the SMscore. Hyperparameters were optimized using machine learning models to achieve the most accurate prognostic model. The potential utility of the SMscore in GC prognosis was evaluated, and hub gene expression was validated through immunohistochemistry (IHC) staining.

**Results:**

*ELOVL4*, *NOS3*, and *ABCA2* were identified as key prognostic genes from a pool of 556 SMRGs. The optimal prognostic model was developed and validated, demonstrating robust predictive performance. IHC staining revealed increased expression of *ELOVL4* and *NOS3* in tumor tissues, which correlated significantly with poor prognosis.

**Conclusion:**

Bioinformatics analysis and IHC validation suggest that *ELOVL4* expression may serve as a prognostic biomarker for GC, providing new insights for prognosis prediction and therapeutic target development in gastric cancer.

## 1 INTRODUCTION

Gastric cancer (GC) ranks as the third most prevalent and fatal cancer in China, representing a substantial proportion of global GC cases and related deaths [1]. Although advances in medical technology have contributed to a decline in GC-related mortality, the disease often lacks distinct clinical manifestations in both early and advanced stages, leading to delayed diagnoses and missed opportunities for timely intervention. Currently, the five-year survival rate for patients with stage IA and IB GC who undergo surgery is 60 to 80%, whereas for those in stage IIB and beyond, the survival rate drops to 35% or lower [2, 3]. Thus, it is crucial to enhance primary and secondary prevention measures for GC and develop novel prognostic biomarkers to guide treatment strategies.

Sphingolipids and their metabolites, essential components of cellular lipids, play pivotal roles in maintaining cell structure and signaling processes. These include regulation of cell barrier function, motility, cycle, adhesion, differentiation, and apoptosis [4]. Ceramide (Cer), an intracellular messenger, plays a key role in regulating cell fate, directly influencing mechanisms of cell growth arrest, differentiation, and aging [5, 6]. Cer accumulation has been shown to promote tumor proliferation while inhibiting apoptosis. Additionally, sphingosine-1-phosphate (S1P) not only promotes cell proliferation but also protects cells from apoptosis; increased S1P synthesis in tumor cells has been linked to the inhibition of apoptosis and the promotion of cell survival [7]. Recent studies have revealed significant differences in lipid metabolism between patients with chronic gastritis and patients with GC, with targeted metabolomics identifying notable upregulation of sphingolipids and Cer in GC tissues (Fold Change (FC) > 1.5) [8]. Furthermore, sphingolipid metabolism-related genes (SMRGs) have demonstrated prognostic potential in cancers, such as oral [9], lung [10], and breast cancer [11].

Despite extensive research into lipid metabolism in GC, previous studies have predominantly focused on general lipid profiles, with limited exploration of Sphingolipid metabolism (SM) from a prognostic perspective. Although several prognostic models have been proposed, these models often lack clinical validation through postoperative patient samples and remain primarily based on cell and animal studies. In contrast, this study employs a comprehensive analysis of clinically collected samples and corresponding data through bioinformatics methods coupled with hyperparameter optimization to refine the prognostic model. Postoperative sample validation further strengthens the reliability and clinical applicability of the findings. This study not only introduces a novel prognostic marker for GC but also provides valuable insights for clinical management and therapeutic strategies.

## 2 MATERIALS AND METHODS

### 2.1 Data Collection

This study encompassed data collection, processing, analysis, and validation. The TCGA-STAD gastric cancer dataset was retrieved from the TCGA database, including 337 GC samples and 30 adjacent normal tissue samples, which were used as the training set. All samples had a survival duration of more than 30 days. Additionally, the GSE84437 gastric cancer dataset was obtained from the GEO database, containing 421 GC samples, all with survival times exceeding 30 days, which served as the validation set. The data were annotated using Perl 3.6 software.

The GeneCards database was queried using the keyword “Sphingolipids” and the filtering criterion “Score > 1.0.” A total of 556 SMRGs were collected for subsequent analysis.

### 2.2 Acquisition of Sphingolipid Metabolism Hub Genes

Initially, the expression profiles of the 556 SMRGs in the TCGA-STAD dataset were examined to assess their expression in both GC and adjacent tissues. Differentially expressed genes (DEGs) were identified using the R package “limma,” with a threshold of |log2 FC| > 0.585 and adj-*P*-value < 0.05, and visualized using a volcano plot. Univariate Cox regression analysis was then performed on the DEGs (*P* < 0.05) using the R packages “survival” and “survminer,” followed by the creation of a forest plot.

Next, Lasso-Cox regression analysis was conducted on survival-associated genes using the R package “glmnet” to identify signature genes. A random forest (RF) model was constructed with the R package “randomForestSRC,” and survival-related genes were scored based on their importance, with scores greater than 0 ranking among the top 10 genes. The intersection of the results from both methods was considered the key survival gene for this study.

Finally, multivariate Cox regression analysis was conducted using the R packages “My.stepwise,” “survival,” and “survminer” to identify hub genes, with the results displayed in a forest plot.

### 2.3 Sphingolipid Metabolism Score and Prognostic Analysis

Stepwise regression was applied to perform multivariate Cox regression analysis, through which the final risk factors were identified, and the regression coefficients for constructing the sphingolipid metabolism score (SMscore) were determined. The SMscore for patients with GC was then established based on the multivariate Cox results and gene expression as follows: SMscore = Σβi * Expi, where Expi represents the expression of the risk gene, and βi denotes the Cox regression coefficient for that gene [12, 13].

The SMscore for each sample in the TCGA-STAD and GSE84437 datasets was subsequently calculated. Based on the median SMscore, the samples were divided into high-SMscore and low-SMscore groups. Expression heat maps, Kaplan-Meier (KM) survival curves, and time-dependent receiver operating characteristic (t-ROC) curves for hub genes were generated for further analysis.

### 2.4 Sphingolipid Metabolism Score and Clinical Characteristics

Clinical data from the TCGA-STAD cohort, including gender, age, and TNM stage, were downloaded and integrated with SMscore and survival time. Statistical comparisons were conducted to assess differences in SMscore across various clinical features.

### 2.5 Clinical Prediction Model

A Lasso-Cox model was constructed using gender, age, TNM stage, and SMscore to identify key prognostic variables. The categorical variables (Table **1**) were encoded for analysis. The simplest model corresponding to λ.1se was selected to extract key prognostic variables. For the TCGA-STAD dataset, the data were split into a training set (70%) and an internal validation set (30%). The training set was utilized for model construction and hyperparameter tuning. Five survival prediction models—gradient boosting machine (GBM), support vector machine (SVM), RF, extreme gradient boosting tree (Xgboost), and Cox proportional hazards model (Coxph)—were constructed. The internal validation set was used to compute the Brier score for each model, which ranges from 0 to 0.25. A Brier score closer to 0 indicates stronger predictive capability, whereas scores exceeding 0.25 suggest limited predictive power. The model with the best performance was evaluated using the GSE84437 dataset.

### 2.6 Sphingolipid Metabolism Score and Immune Landscape

CIBERSORT, a deconvolution algorithm, was employed to analyze the immune cell composition in the TCGA-STAD dataset for the high-SMscore and low-SMscore groups based on the input reference gene set.

Single-sample gene set enrichment analysis (ssGSEA) was conducted on individual samples. The gene list ranking and enrichment score (ES) calculation were based on the expression levels of genes within each sample rather than correlations between genes and phenotypes. Immune function expression analysis was performed on samples from each group using the reference gene set “immune.gmt,” and differences in immune function were compared between the groups.

Lastly, the expression of common immune checkpoint genes in each group was obtained, and their correlations with hub genes and SMscore were calculated and visualized using the R packages “Corrplot” and “ggplot2.”

### 2.7 Gene Set Enrichment Analysis and Tumor Mutational Burden Analysis

Gene set enrichment analysis (GSEA) was performed on the high and low SMscore groups to identify differentially expressed Kyoto Encyclopedia of Genes and Genomes (KEGG) signaling pathways (*P* < 0.05), with the top five significant results being reported.

Mutation expression data for the TCGA-STAD dataset were downloaded and annotated using Perl 3.6 software. Survival data for the high-SMscore and low-SMscore groups were extracted, and tumor mutation burden (TMB) differences between the two groups were assessed, along with their correlation with SMscore. The TMB index was divided into high and low categories based on the median value, and KM survival curves were plotted for the two groups according to their SMscore.

### 2.8 Potential Drug Prediction

The drug sensitivity of patients with GC was predicted using data from the Genomics of Drug Sensitivity in Cancer (GDSC) database. The half maximal inhibitory concentration (IC50) values were calculated using the R package “pRRophetic” to evaluate drug reactivity.

### 2.9 Clinical Sample Collection

From June 2011 to June 2017, clinical data from 180 patients with GC who underwent radical gastrectomy at Baoding First Central Hospital were collected, along with postoperative pathology results. Patients with a prior history of other cancers were excluded. GC tissue specimens were obtained from paraffin blocks stored in the Department of Pathology, Baoding First Central Hospital, and diagnoses were independently confirmed by two professional pathologists. Ethical approval was granted by the Ethics Committee of Baoding First Central Hospital (Ethics Approval number: (2024)56).

### 2.10 Immunohistochemical Staining and Image Analysis

Tissue chips were placed in a 65°C constant-temperature drying oven for 1 hour, followed by soaking in xylene for 30 minutes and anhydrous ethanol for 20 minutes. After rinsing with distilled water and PBS, antigen retrieval and endogenous peroxidase blocking were performed. Primary and secondary antibodies were then applied, and the tissues underwent color development, counterstaining, dehydration, transparency, and encapsulation.

Immunohistochemistry (IHC) results were independently evaluated by two senior pathologists using a semi-quantitative method. Five representative areas were randomly selected from a 200x magnification field under a microscope. The proportion of positive cells was scored as follows: ≤ 9% = 0 points; 10-25% = 1 point; 26-50% = 2 points; 51-75% = 3 points; 76-100% = 4 points. The staining intensity was scored as follows: 0 points for no staining, 1 point for light yellow, 2 points for yellow, and 3 points for brown. The immune response score was calculated by multiplying the two scores, with scores of 0-4 indicating no/low expression and scores of 6-12 indicating high expression.

### 2.11 Statistical Analysis

All statistical analyses were performed using R software (version 4.3.1). The results were statistically significant when the *P*-value was under 0.05.

## 3 RESULTS

### 3.1 Characteristic Sphingolipid Metabolism Genes

In this study, 532 SMRGs were identified. In the TCGA-STAD dataset, 190 DEGs in SM were filtered out (Fig. **1A**). Univariate Cox analysis identified 22 survival-associated genes (Fig. **1B**). Lasso-Cox analysis was performed, resulting in the identification of 13 key survival-related genes (Fig. **1C**). RF analysis revealed the top 10 genes closely associated with survival (Fig. **1D**). Ultimately, five key survival genes were selected: *ELOVL4*, *NOS3*, *ABCA2*, *TRAF2*, and *MSX2* (Fig. **1E**). By applying multivariate Cox analysis, three hub genes were identified: *ELOVL4*, *NOS3*, and *ABCA2* (Fig. **1F**).

### 3.2 Sphingolipid Metabolism Score and Prognostic Characteristics

The SMscore for each sample was calculated using the multivariate Cox model: SMscore = *ELOVL4* * 0.3379 + *NOS3* * 0.4224 - *ABCA2* * 0.4709. Higher SMscores were associated with shorter survival times (Fig. **2A**). KM survival analysis revealed a significant difference in prognostic outcomes between the high-SMscore and low-SMscore groups (Fig. **2B**). The areas under the curve (AUCs) for SMscore in predicting 1, 3, and 5-year survival in the TCGA-STAD dataset were 0.66, 0.71, and 0.68, respectively. For the GSE84437 dataset, the AUC values were 0.60, 0.57, and 0.62, respectively (Fig. **2C**).

### 3.3 Sphingolipid Metabolism Score and Clinical Characteristics

Analysis of various clinical features showed few differences between the two groups (*P* > 0.05) (Fig. **3A**). However, SMscores for individuals under 65 years old were significantly lower than those for individuals over 65 years (*P* = 0.025) (Fig. **3B**). No significant differences in SMscore were observed across TNM stages (*P* > 0.05) (Figs. **3C**-**3E**).

### 3.4 Best Prognostic Model

A Lasso-Cox model incorporating gender, age, TNM stage, and SMscore identified N-stage and SMscore as the key prognostic factors in GC (Fig. **4A**). Hyperparameter tuning was performed for the model (Figs. **4B**-**4E**). The Xgboost model, with a Brier score exceeding 0.25, was excluded, and the remaining models, including Coxph, exhibited Brier scores below 0.25 (Fig. **4F**). Since Coxph, as a traditional prognostic model, demonstrated strong interpretability and accuracy comparable to that of the machine learning models, it was selected as the best prognostic model. In the GSE84437 dataset, the AUCs for the Coxph model at 1, 3, and 5 years were 0.57, 0.59, and 0.65, respectively (Fig. **4G**). This model outperformed the SMscore-based t-ROC for 3 and 5-year survival predictions. Finally, the model was visualized as a Nomogram (Fig. **4H**).

### 3.5 Immune Infiltration Analysis

The high SMscore group exhibited significantly lower percentages of CD4^+^ T cell memory and NK cell resting (*P* < 0.05), while monocytes (*P* < 0.01), M2 macrophages (*P* < 0.05), resting dendritic cells (*P* < 0.01), and resting mast cells (*P* < 0.01) were significantly elevated in comparison (Fig. **5A**). Immune function analysis revealed substantial activation of the high SMscore group in APC co-stimulation (*P* < 0.05), CCR (*P* < 0.001), HLA (*P* < 0.01), Parainflammation (*P* < 0.01), Type I IFN response (*P* < 0.01), and Type II IFN response (*P* < 0.001) (Fig. **5B**). Furthermore, SMscore and the three hub genes were strongly associated with immune checkpoint-related genes, with *NRP1*, *ADORA2A*, *TNFRSF8*, and *TNFRSF9* showing positive correlations (Fig. **5C**).

### 3.6 GSEA and TMB Analyses

In the context of GC, the high-SMscore group was predominantly enriched in KEGG pathways related to vascular smooth muscle contraction and calcium signaling (Fig. **6A**), while the low-SMscore group exhibited higher enrichment in pathways, such as cell cycle, spliceosome, and pyrimidine metabolism.

The mutation score was notably higher in the low SMscore group, suggesting a greater susceptibility to tumor-targeting agents (Figs. **6B**-**6C**). A significant survival difference was observed between the two TMB groups, with patients possessing low mutation scores demonstrating shorter survival times (Fig. **6D**). Furthermore, the poorest prognosis was observed in the low mutation, high-SMscore subgroup (Fig. **6E**).

### 3.7 Potential Therapeutic Agents

Patients with GC in the low-SMscore group showed higher sensitivity to Bortezomib, Dasatinib, Imatinib, Sunitinib, and Pazopanib (*P* < 0.001), whereas the high-SMscore group exhibited greater sensitivity to Afatinib (*P* < 0.001) (Figs. **7A**-**7F**).

### 3.8 Results of IHC Experiments

GC tissue analysis yielded the following results: the expression of *ELOVL4* (Figs. **8A**-**8C**), *NOS3* (Figs. **8D**-**8F**), and *ABCA2* (Figs. **8G**-**8I**) was categorized into high, low, and non-expression groups.

### 3.9 Prognostic Analysis

The survival data for GC were integrated with the expression levels of *ELOVL4*, *NOS3*, and *ABCA2* for KM survival analysis. A significant divergence in survival time was observed for *ELOVL4* and *NOS3* expressions (Figs. **9A**-**9B**), while no significant difference in survival was found for *ABCA2* expression (Fig. **9C**).

Further analysis using univariate Cox regression identified *ELOVL4* expression, *NOS3* expression, *ABCA2* expression, and TNM stage as potential risk factors. Multivariate Cox regression analysis revealed that *ELOVL4* expression and TNM stage were independent prognostic factors for GC (*P* < 0.05) (Table **2**).

## 4 DISCUSSION

GC is a prevalent malignant tumor, with an incidence exceeding 800,000 new cases annually, primarily concentrated in Asia and particularly in China, which records the highest number of new GC cases. The lack of specific clinical symptoms, coupled with the high heterogeneity of GC and a significant recurrence rate after treatment, contributes to an increasing mortality rate. The five-year survival rate for patients with GC remains below 30%, posing a major challenge to China’s healthcare system [14, 15]. Thus, the primary goal of this study is to identify biomarkers that can predict GC prognosis, develop novel therapeutic targets, and provide innovative diagnostic and treatment strategies.

Recent studies have reported that patients with GC exhibit the upregulation of 357 lipid metabolism-related genes and the downregulation of 185 lipid metabolism genes compared to healthy individuals, indicating a significant reprogramming of lipid metabolism in patients with GC [16]. This metabolic reprogramming has profound effects on the tumor immune microenvironment, playing a critical role in both immune cell and tumor cell survival and proliferation. Additionally, lipid metabolism is crucial in regulating the proliferation, invasion, and metastasis of tumor cells [17]. Sphingolipid metabolism, an essential component of lipid metabolism, has attracted growing attention in cancer research in recent years. Key enzymes and metabolites in the sphingolipid metabolic pathway, particularly S1P, are widely distributed in the tumor microenvironment. S1P, a critical sphingolipid metabolite, is produced through the phosphorylation of sphingosine by sphingosine kinases (SphK1 and SphK2) [18]. S1P in cells not only serves as a cofactor for binding histones, nuclear factor-κB (NF-κB), and other molecules [19-21] but also activates multiple signaling pathways [22-24], thereby regulating gene expression and promoting anti-apoptotic responses. Numerous studies have demonstrated elevated levels of S1P and SphK1 in various tumor tissues, including GC [25] and colorectal cancer [26]. SphK1 has also been identified as an oncogene with significant tumorigenic potential [27]. While the relationship between SM and GC is complex, limited research has been conducted on its prognostic implications in GC.

This study utilized bioinformatics approaches to explore and validate the association between SMRGs and GC prognosis, offering a novel perspective on GC prognosis and treatment from the lens of SM [28]. Initially, relevant data were extracted from the TCGA-STAD and GSE84437 datasets, which were cleaned and processed. SMRGs were identified using the GeneCards database, and differential expression analysis was conducted. Subsequently, three hub genes—*ELOVL4*, *NOS3*, and *ABCA2*—were selected through multiple survival analyses. The SMscore was calculated based on the hub genes. After performing hyperparameter tuning, the Coxph model emerged as the optimal prognostic model, which was then validated.

The immune landscape within the tumor microenvironment plays a pivotal role in GC prognosis. Previous studies have demonstrated that alterations in immune cell composition and function significantly impact the prognosis of GC [28, 29]. In this study, the immune cell composition and immune function of two groups—divided based on the median SMscore—were analyzed.

Gene mutations in tumors generate neoantigens that are detected by the immune system, making tumor gene mutations a key consideration for immunotherapy [30]. The prediction of immunotherapy effectiveness often incorporates tumor mutational burden (TMB) [31, 32]. Growing evidence suggests that tumors with higher TMB are more likely to benefit from immune checkpoint inhibitor (ICI)-based therapies [33]. By analyzing genes associated with immune checkpoints, this study further revealed that *NRP1*, *ADORA2A*, *TNFRSF8*, and *TNFRSF9* were strongly correlated with the selected key genes and the prognostic model developed herein.

Among the three hub genes screened, *ELOVL4* plays a critical role in the synthesis of long-chain fatty acids. Fatty acids are essential for the expansion, metastasis, and energy production of tumor cells in the dynamic tumor microenvironment. Tumor cells can accelerate fatty acid metabolism (FAM) to supply energy and sustain rapid growth by remodeling lipid metabolic pathways [34, 35]. Czumaj *et al.* demonstrated that silencing *ELOVL4* effectively inhibited the rate of tumor cell proliferation and migration in colorectal cancer [36]. Similarly, altered *ELOVL4* expression has been linked to poorer survival outcomes in bladder cancer [37].

*NOS3*, also known as endothelial nitric oxide synthase (eNOS), is an essential enzyme in sphingolipid signaling. S1P has been shown to activate eNOS by binding to endothelial differentiation gene receptors. Additionally, S1P can activate eNOS through G protein-coupled receptors, triggering the PI3-K/Akt pathway and resulting in the phosphorylation of serine residues in eNOS. Therefore, sphingolipid metabolism is closely associated with *NOS3* regulation [38, 39]. Elevated expression of *NOS3* has been observed in various tumor tissues, including breast cancer (BC), pancreatic cancer, GC, and colorectal cancer, where it plays a pivotal role in tumor growth, invasion, and angiogenesis [40]. Furthermore, studies have shown that reduced miR-605-3p expression is an independent risk factor for poor GC prognosis. This downregulation leads to the upregulation of VAMP3, which increases the release of NOS3 through exosomes, promoting local vascular formation and enhancing GC cell metastasis [41].

In this study, bioinformatics analyses and clinical experiments validated the involvement of *ELOVL4* and *NOS3* in GC. It was found that both factors are associated with reduced survival in patients with GC, with ELOVL4 expression emerging as an independent risk factor for GC prognosis.

The *ABCA2* gene is a member of the A family within the ATP-binding cassette (ABC) superfamily of transmembrane proteins, primarily encoding ABC transporters. A key factor contributing to the failure of many tumor treatment strategies is drug resistance, which often arises due to the active efflux of cytotoxic substances *via* ABC transporter-mediated ATP hydrolysis [42]. For example, *ABCA2* protein has been implicated in multidrug resistance in cancers, such as lung, breast, and endometrial cancers [43, 44]. Additionally, the overexpression of ABC transporters has a complex relationship with drug resistance in cancers like colorectal and renal cancers [45, 46]. The expression of specific ABC transporters is also linked to cancer prognosis, with numerous studies indicating that the overexpression of certain transporters is associated with reduced survival in neuroblastoma and breast cancer [47]. Moreover, whole-genome sequencing of tumor tissue samples and matched normal tissues from solitary fibrous tumors/hemangiopericytomas (SFT/HPC) has revealed missense mutations in the ABCA2 gene, which may contribute to its pathogenic potential [48]. However, in this study, *ABCA2* expression was not found to be associated with GC prognosis, which contradicts the findings from bioinformatics analyses. This discrepancy suggests that in GC, the expression of ABC transporters may be attributed to other subfamilies within the ABC superfamily (A-G). Given that many patients with GC are diagnosed at advanced stages, chemotherapy becomes particularly crucial in their treatment. Therefore, further investigation into the specific types of ABC transporters expressed in GC, along with the mutation profiles of their corresponding coding genes, could facilitate the development of effective ABC transporter inhibitors. These inhibitors, when combined with chemotherapy agents, could improve therapeutic efficacy and patient prognosis in GC treatment.

## 5 LIMITATION

This project not only develops an optimal prognostic model through hyperparameter optimization but also validates the model using IHC analysis of clinical postoperative samples, thereby ensuring the reliability of the results. However, a limitation of this study is the inconsistency between the IHC validation and bioinformatics analysis results for the *ABCA2* gene. Additionally, the relationship between the ABC gene superfamily and GC has not been thoroughly explored. Therefore, further investigation into this aspect is necessary to refine the understanding and improve the model in future studies.

## CONCLUSION

In summary, the prognostic model developed in this study, based on three SMRGs, demonstrates a strong ability to predict the prognosis of patients with GC. Clinical validation using postoperative samples confirmed by IHC revealed that *ELOVL4* and *NOS3* were highly expressed in GC tissues associated with poor prognosis. Notably, *ELOVL4* emerged as a promising new prognostic marker for GC, offering the potential as a valuable tool in guiding immunotherapy strategies for GC treatment.

## Figures and Tables

**Fig. (1):**
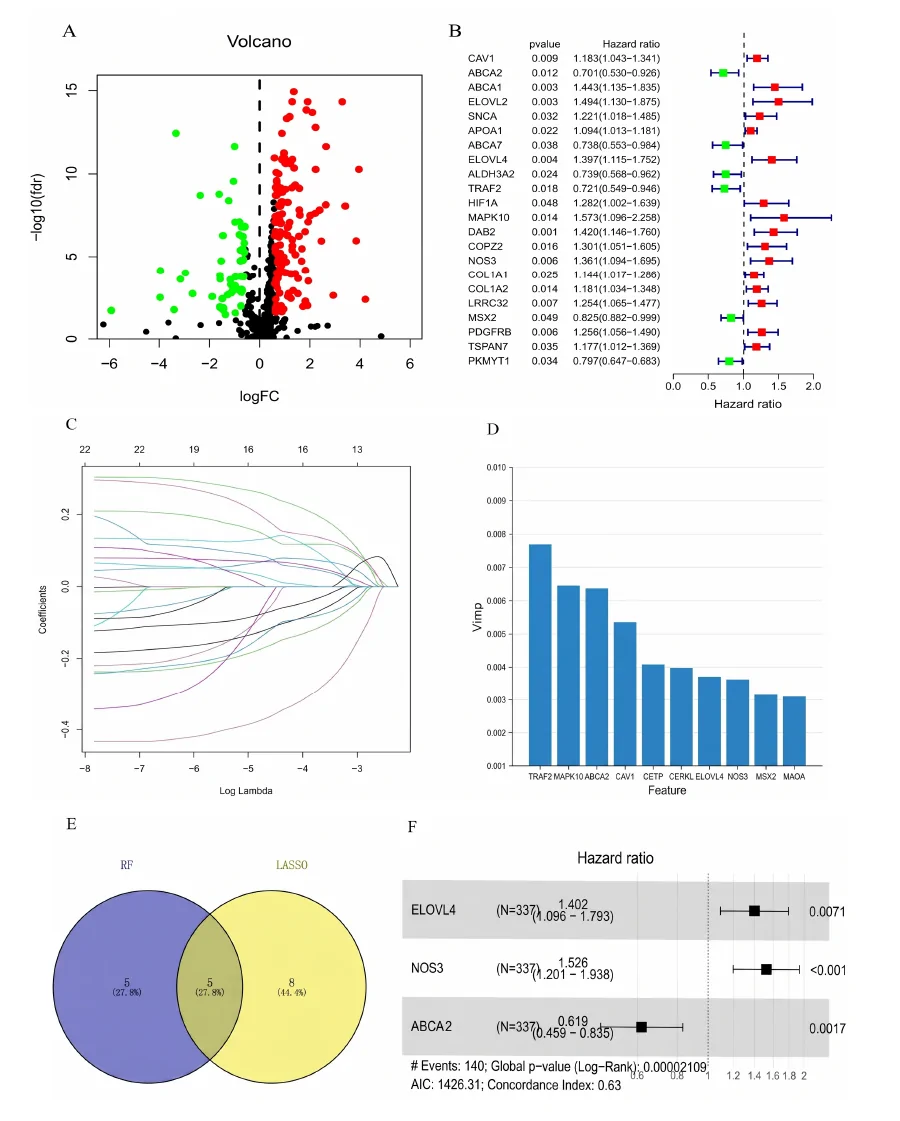
Feature gene screening. **(A)** Differentially expressed SMRGs. **(B)** Univariate COX regression analysis. **(C)** Feature genes were screened based on Lasso-cox analysis. **(D)** Based on random survival forest characteristic gene score. **(E)** Key survival genes were obtained by intersection. **(F)** The hub genes were obtained by Multivariate Cox regression analysis.

**Fig. (2):**
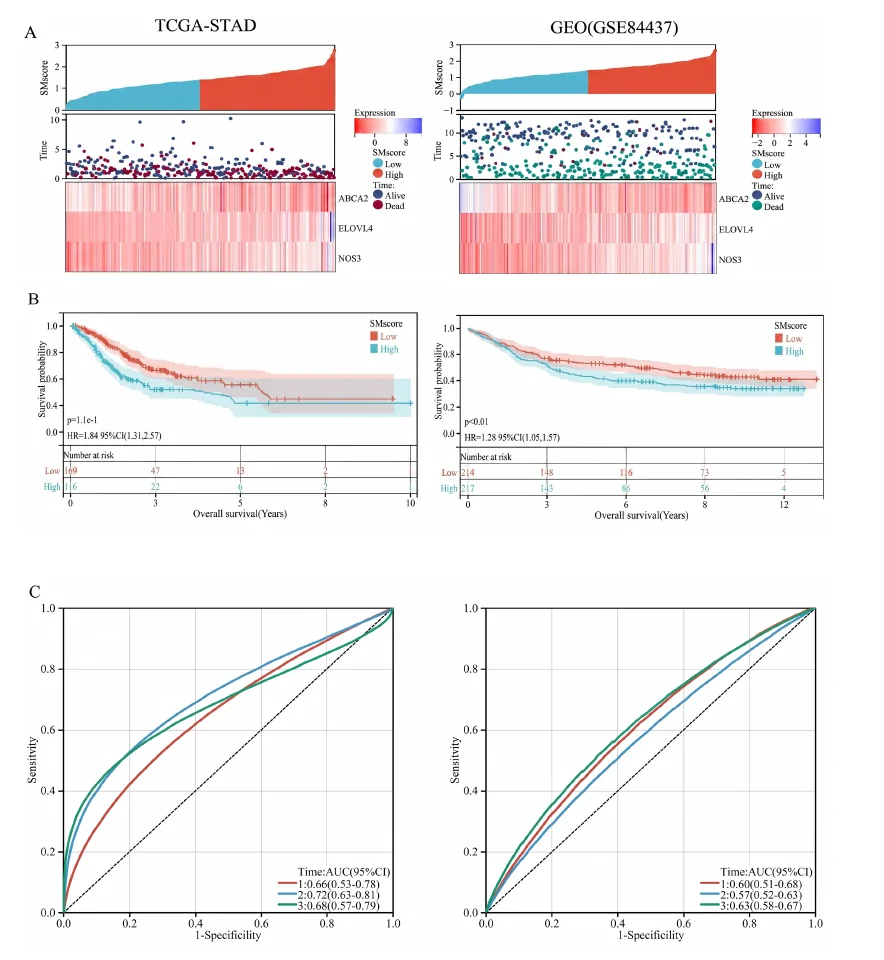
SMscore in TCGA-STAD and GSE84437 prognostic analysis. **(A)** Overall survival time and hub gene expression of samples in high- and low-SMscore groups. **(B)** KM survival curve of high- and low-SMscore group. **(C)** t-ROC in the TCGA-STAD database and the GSE84437.

**Fig. (3):**
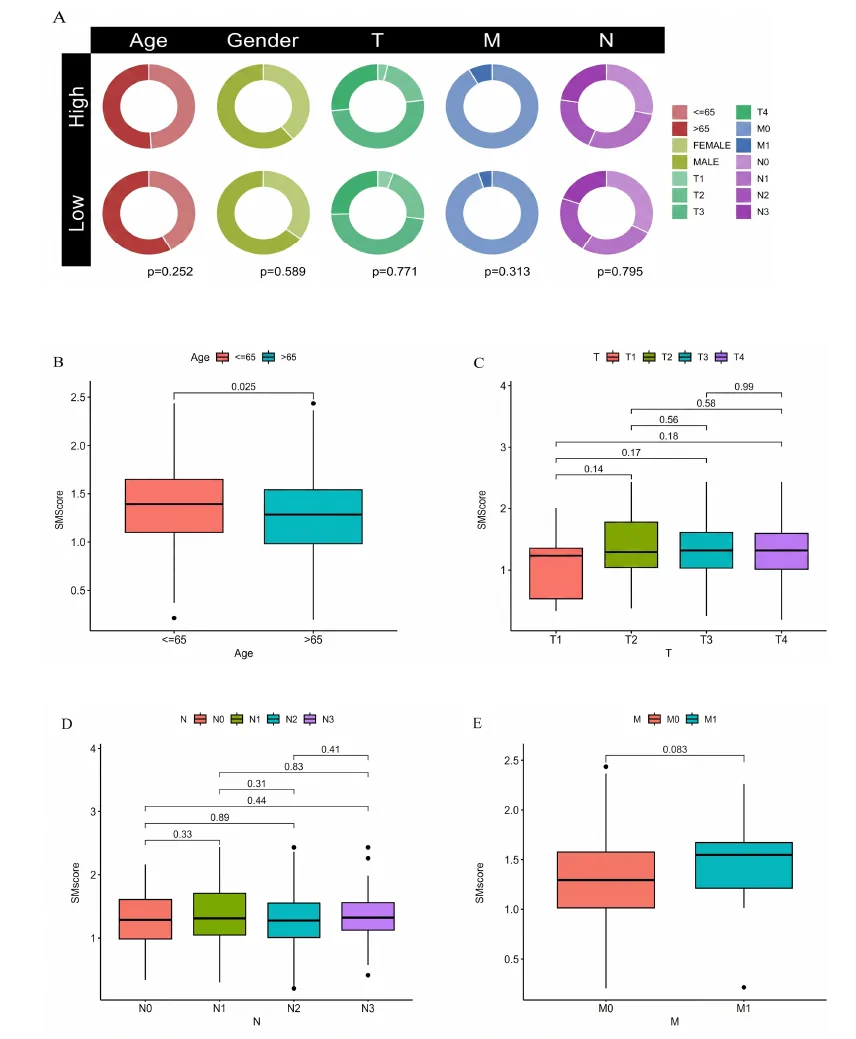
Analysis of SMscore and clinical characteristics. **(A)** Clinical information of high-and low-SMscore groups. **(B-E)** SMscore was different in Age and TNM stage.*(A higher resolution / colour version of this figure is available in the electronic copy of the article)*.

**Fig. (4):**
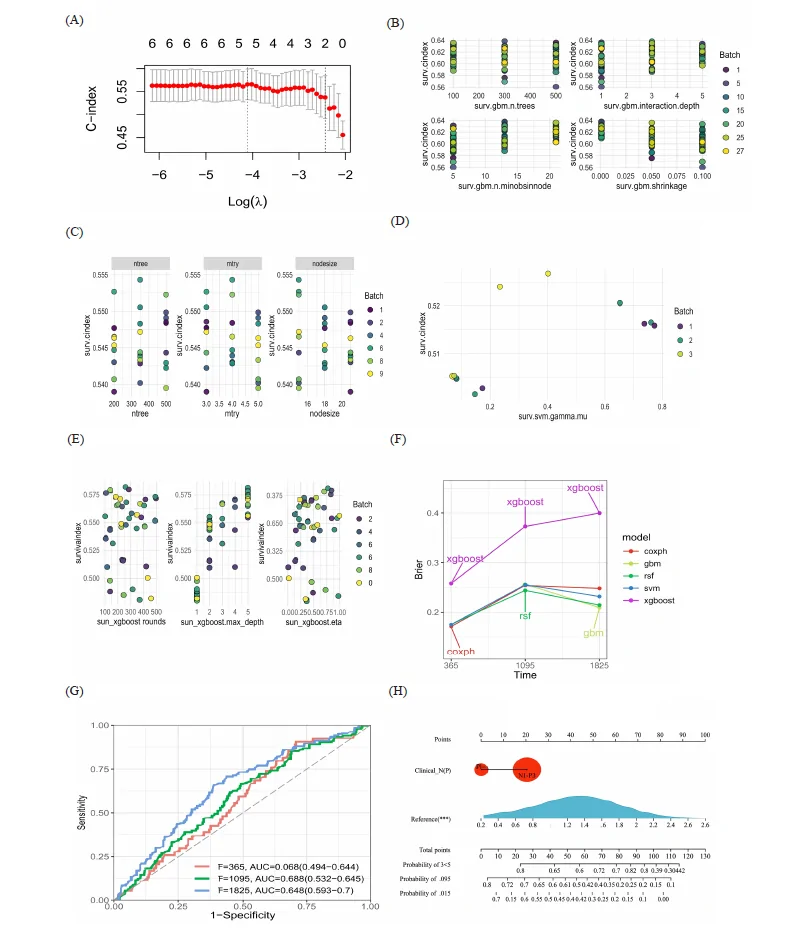
Optimal prognosis model screening. **(A)** Identified key prognostic variables. **(B-E)** Hyperparameter tuning of machine learning models. F Brier score. **(G)** Model performance in an external validation set. **(H)** Visualization of the model.

**Fig. (5):**
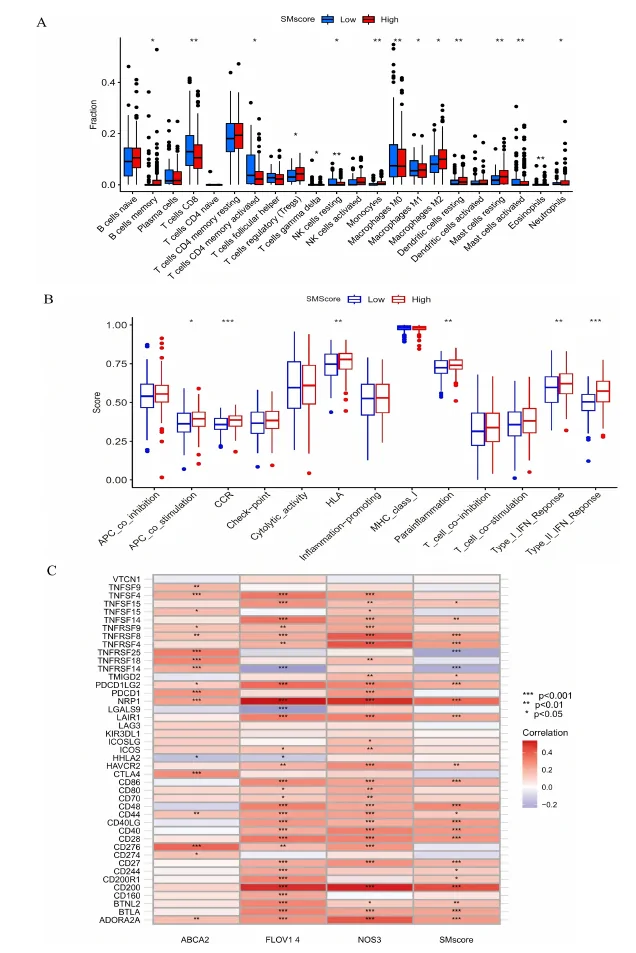
Immune infiltration analysis. **(A)** Difference analysis of 22 immune cell scores between high- and low-SMscore groups. **(B)** Analysis of differences in immune pathway scores between high- and low-SMscore groups. **(C)** Correlation analysis between hub genes, immune scores, and immune checkpoints.

**Fig. (6):**
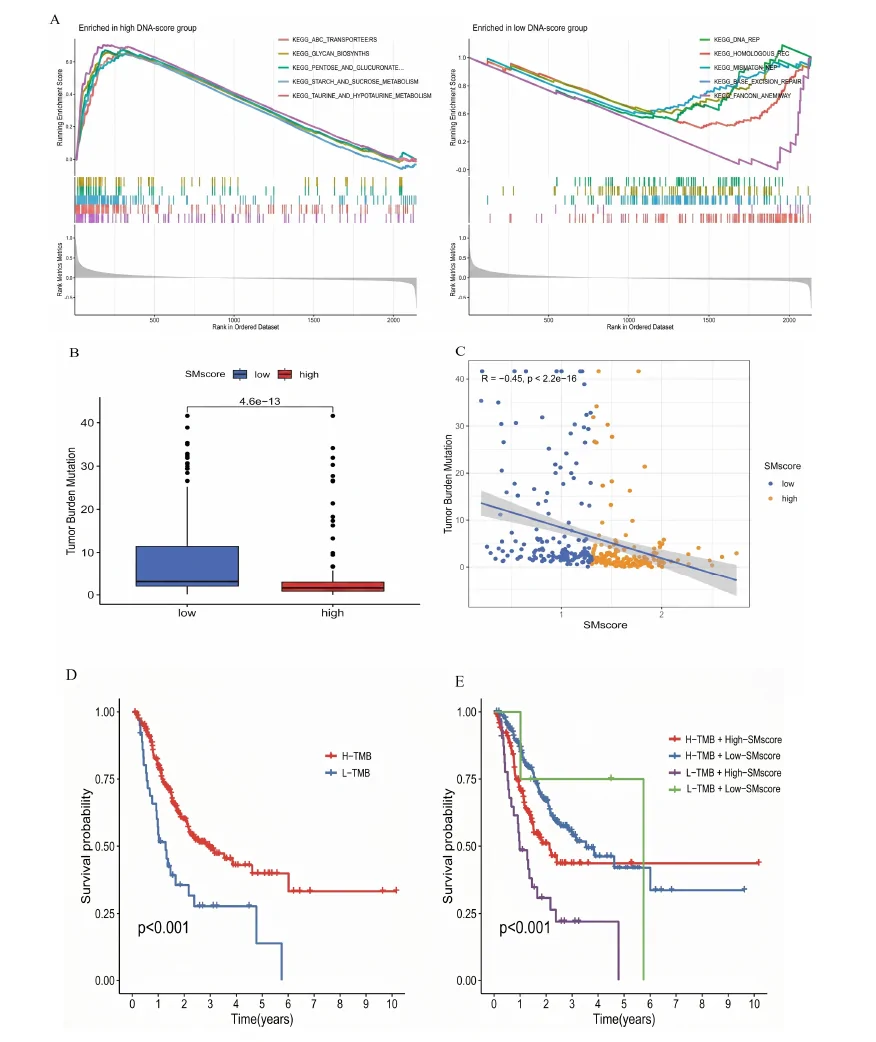
GSEA and TMB analysis. **(A)** Main enriched pathways in the high- and low-SMscore groups. **(B-C)** The correlation between SMscore and TMB was analyzed. **(D)** KM survival curves for high and low TMB score groups. **(E)** Prognostic KM survival curve of SMscore combined with TMB score.

**Fig. (7):**
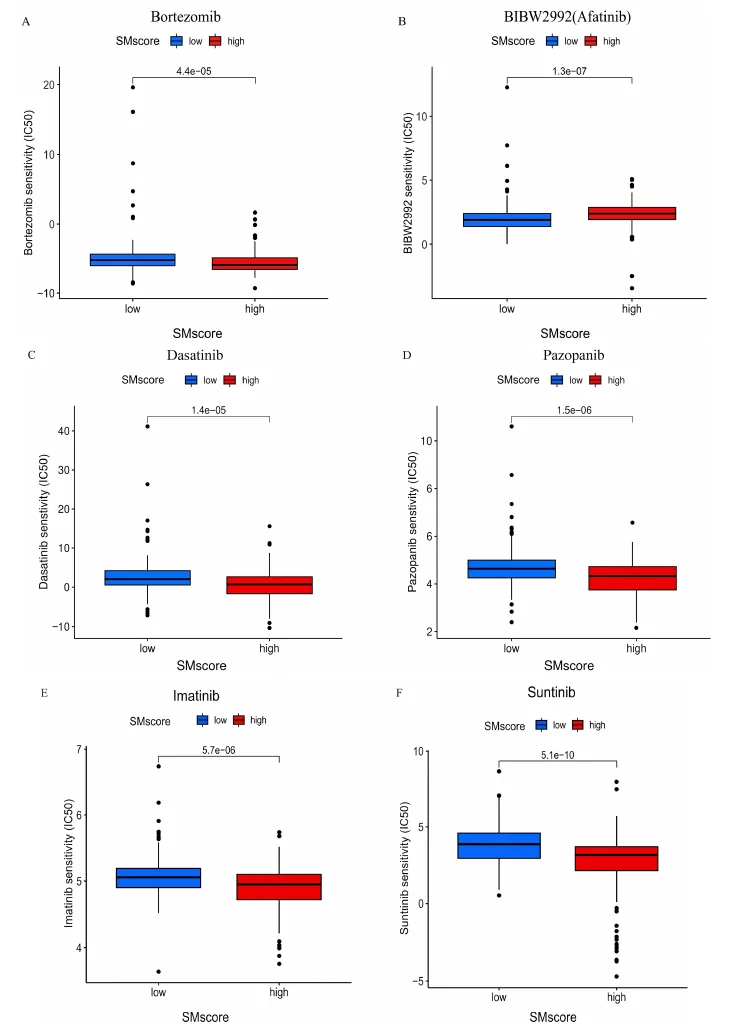
Potential therapeutic drug predictions. **(A)** Bortezomib. **(B)** BIBW2992 (Afatinib). **(C) **Dasatinib. **(D)** Pazopanib. **(E)** Imatinib. **(F)** Suntinib.

**Fig. (8):**
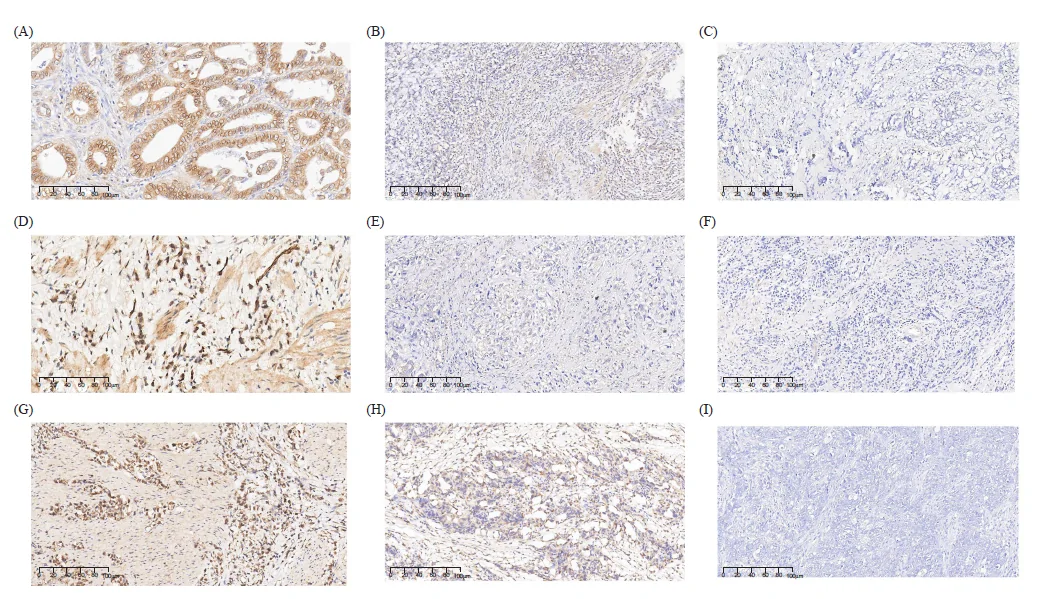
200-fold expression of *ELOVL4*, *NOS3*, and *ABCA2* in GC tissues. **(A-C)** High expression, low expression, and no expression of *ELOVL4*. **(D-F)** High expression, low expression, and no expression of *NOS3*. **(G-I)** High expression, low expression, and no expression of *ABCA2*.

**Fig. (9):**
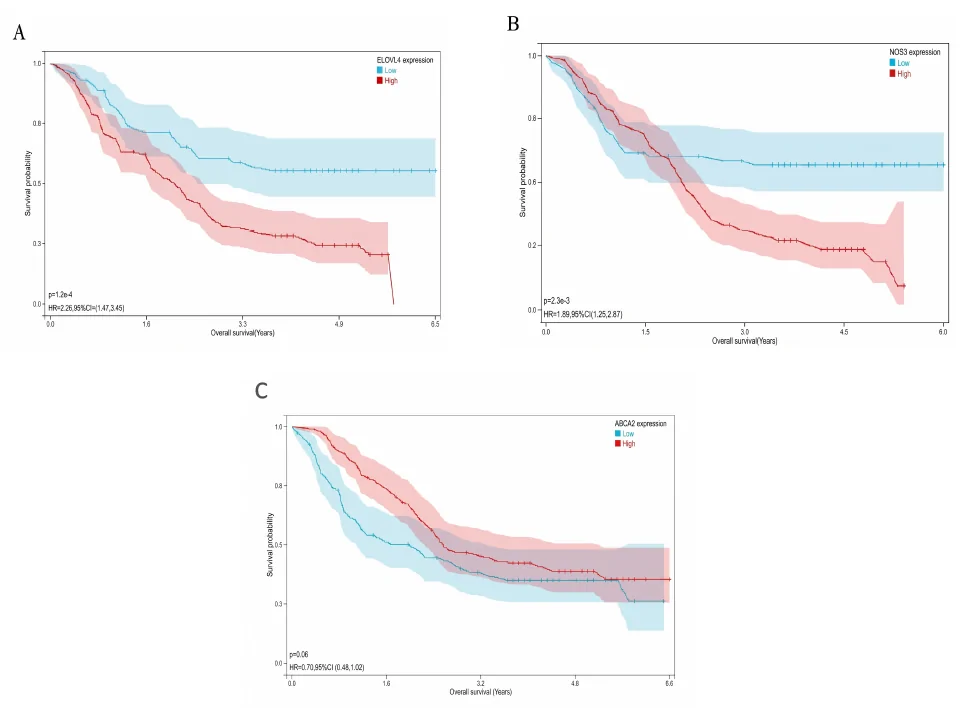
KM survival analysis. **(A)** *ELOVL4* expression. **(B)** *NOS3* expression. **(C)** *ABCA2* expression.

**Table 1:** Coding of categorical variables.

**Categorical Variables**	**Coding**
Gender	Female:0, Male:1
T stage	T1/T2:0, T3/T4:1
N stage	N0:0, N1/N2/N3:1
M stage	M0:0, M1:1

**Note:** The T1/T2 stages are coded as 0. The T3/T4 stages are coded as 1. The N1/N2/N3 stages are coded as 1.

**Table 2:** Independent prognostic analysis.

**Variables**	**Univariate Cox Regression Analyses**					**Multivariate Cox Regression Analyses**				
	**β**	**S.E**	**Z**	***P* value**	**HR (95%CI)**	**β**	**S.E**	**Z**	***P* value**	**HR (95%CI)**
Age	0.01	0.01	1.28	0.199	1.01 (0.99 ~ 1.03)	-	-	-	-	-
Gender	-	-	-	-	-	-	-	-	-	-
Male	-	-	-	-	1.00 (Reference)	-	-	-	-	-
Female	0.04	0.21	0.20	0.839	1.04 (0.70 ~ 1.56)	-	-	-	-	-
Stage	-	-	-	-	-	-	-	-	-	-
Stage1/2	-	-	-	-	1.00 (Reference)	-	-	-	-	1.00 (Reference)
Stage3/4	1.37	0.29	4.73	<0.001*	3.95 (2.24 ~ 6.98)	1.28	0.30	4.27	<0.001*	3.61 (2.00 ~ 6.51)
Lauren	-	-	-	-	-	-	-	-	-	-
Mixed type	-	-	-	-	1.00 (Reference)	-	-	-	-	-
Intestinal type	0.61	0.52	1.18	0.239	1.84 (0.67 ~ 5.07)	-	-	-	-	-
Diffuse type	0.71	0.52	1.36	0.173	2.04 (0.73 ~ 5.68)	-	-	-	-	-
*ELOVL4*	-	-	-	-	-	-	-	-	-	-
Low	-	-	-	-	1.00 (Reference)	-	-	-	-	1.00 (Reference)
High	0.81	0.22	3.75	<0.001*	2.25 (1.47 ~ 3.44)	0.69	0.23	3.01	0.003*	2.00 (1.27 ~ 3.14)
*NOS3*	-	-	-	-	-	-	-	-	-	-
Low	-	-	-	-	1.00 (Reference)	-	-	-	-	1.00 (Reference)
High	0.63	0.21	2.99	0.003*	1.88 (1.24 ~ 2.86)	0.07	0.23	0.32	0.748	1.08 (0.68 ~ 1.70)
*ABCA2*	-	-	-	-	-	-	-	-	-	-
Low	-	-	-	-	1.00 (Reference)	-	-	-	-	-
High	-0.35	0.19	-1.83	0.067	0.70 (0.48 ~ 1.03)	-	-	-	-	-

**Note:** β: partial regression coefficient, S.E: standard error of the regression coefficient, Z: Wald stats, HR: Hazard Ratio, CI: Confidence Interval *P>0.05

## Data Availability

The data that support the findings of this study are available on request from the corresponding author, Hui-Qing Zhang, upon reasonable request.

## LIST OF ABBREVIATIONS

BC: Breast Cancer
Cer: Ceramide
Coxph: Cox Proportional Hazards Model
ES: Enrichment Score
FC: Fold Change
GBM: Gradient Lifting Machine
GC: Gastric Cancer
GDSC: Genomics of Drug Sensitivity in Cancer
IC50: Half Maximal Inhibitory Concentration
IHC: Immunohistochemistry
KEGG: Kyoto Encyclopedia of Genes and Genomes
RF: Random Forest
S1P: Sphingosine-1-phosphate
SM: Sphingolipid Metabolism
SMRGs: Sphingolipid Metabolism-related Genes
SMscore: Sphingolipid Metabolism Score
ssGSEA: Single Sample Gene set Enrichment Analysis
SVM: Support Vector Machine
TMB: Tumor Mutation Burden
t-ROC: time-dependent Receiver Operating Curve
Xgboost: Extreme Gradient Boosting Tree
